# The Evolution of Orphan Regions in Genomes of a Fungal Pathogen of Wheat

**DOI:** 10.1128/mBio.01231-16

**Published:** 2016-10-18

**Authors:** Clémence Plissonneau, Alessandra Stürchler, Daniel Croll

**Affiliations:** a Plant Pathology, Institute of Integrative Biology, ETH Zurich, Zürich, Switzerland; b UMR BIOGER, INRA, AgroParisTech, Université Paris-Saclay, Saint-Aubin and Thiverval-Grignon, France

## Abstract

Fungal plant pathogens rapidly evolve virulence on resistant hosts through mutations in genes encoding proteins that modulate the host immune responses. The mutational spectrum likely includes chromosomal rearrangements responsible for gains or losses of entire genes. However, the mechanisms creating adaptive structural variation in fungal pathogen populations are poorly understood. We used complete genome assemblies to quantify structural variants segregating in the highly polymorphic fungal wheat pathogen *Zymoseptoria tritici*. The genetic basis of virulence in *Z. tritici* is complex, and populations harbor significant genetic variation for virulence; hence, we aimed to identify whether structural variation led to functional differences. We combined single-molecule real-time sequencing, genetic maps, and transcriptomics data to generate a fully assembled and annotated genome of the highly virulent field isolate 3D7. Comparative genomics analyses against the complete reference genome IPO323 identified large chromosomal inversions and the complete gain or loss of transposable-element clusters, explaining the extensive chromosomal-length polymorphisms found in this species. Both the 3D7 and IPO323 genomes harbored long tracts of sequences exclusive to one of the two genomes. These orphan regions contained 296 genes unique to the 3D7 genome and not previously known for this species. These orphan genes tended to be organized in clusters and showed evidence of mutational decay. Moreover, the orphan genes were enriched in genes encoding putative effectors and included a gene that is one of the most upregulated putative effector genes during wheat infection. Our study showed that this pathogen species harbored extensive chromosomal structure polymorphism that may drive the evolution of virulence.

## IMPORTANCE

Pathogen outbreak populations often harbor previously unknown genes conferring virulence. Hence, a key puzzle of rapid pathogen evolution is the origin of such evolutionary novelty in genomes. Chromosomal rearrangements and structural variation in pathogen populations likely play a key role. However, identifying such polymorphism is challenging, as most genome-sequencing approaches only yield information about point mutations. We combined long-read technology and genetic maps to assemble the complete genome of a strain of a highly polymorphic fungal pathogen of wheat. Comparisons against the reference genome of the species showed substantial variation in the chromosome structure and revealed large regions unique to each assembled genome. These regions were enriched in genes encoding likely effector proteins, which are important components of pathogenicity. Our study showed that pathogen populations harbor extensive polymorphism at the chromosome level and that this polymorphism can be a source of adaptive genetic variation in pathogen evolution.

## INTRODUCTION

Eukaryotic genomes evolve by the insertion, deletion, rearrangement, or acquisition of chromosomal sequences (1). Structural changes in chromosomes are linked to key steps in the differentiation of sex chromosomes (2, 3), incipient speciation (4), and the emergence and maintenance of phenotypes (5). Structural variants, in particular inversions, segregating in randomly mating populations have long been thought to underlie key adaptive processes, such as the onset of reproductive isolation or the maintenance of clusters of coadapted genes (6).

Rapid evolution of chromosomal structure is particularly striking among species of plant-pathogenic fungi and oomycetes (7). Through their immune systems, plants exert strong selection pressure on pathogen populations (8, 9). Secreted effector proteins able to interfere with host sensor proteins and counteract the basal and induced defense mechanisms of the host are major components of pathogen virulence. The rapid coevolution between hosts and pathogens leads the host to evolve receptors that specifically detect effector proteins and trigger defense activation. Hence, pathogens expressing detected effectors (i.e., avirulence effectors) suffer a strong fitness penalty in host populations expressing the corresponding receptor. In turn, pathogen populations can rapidly escape host recognition through selection favoring mutations that enable effectors to escape recognition or selection favoring effector gene deletion (10, 11).

Comparative genomics of pathogens shows that genes encoding effectors are not randomly distributed in the genome. Many plant pathogen genomes are compartmentalized into gene-dense regions and gene-poor regions rich in transposable elements (7, 12). Such genome compartmentalization in plant pathogens has been called the “two-speed genome.” Genes encoding putative effectors were found to be enriched in the gene-poor compartments, which show higher rates of evolution. In the *Brassica*-infecting phoma stem canker pathogen *Leptosphaeria maculans*, the genome has experienced a recent and massive invasion of transposable elements that led to two distinct types of isochores (13, 14). The GC isochores are gene dense, while the AT-rich isochores only contain 5% of the predicted genes and are mainly composed of a mosaic of transposable elements degenerated by repeat-induced point mutations (RIPs). RIP is a genomic defense mechanism that prevents the spread of transposable elements by mutating copies of identical sequences (15). AT-rich isochores are enriched in pathogenicity-related genes and can evolve rapidly due to RIPs (16–18). The massively expanded genomes of the closely related species *Phytophthora infestans* (potato late blight pathogen), *Phytophthora ipomoeae*, and *Phytophthora mirabilis* contain large numbers of gene-poor and repeat-rich compartments that are enriched in effector genes (19). These compartments evolve at higher rates, both accumulating point mutations faster and having a propensity to undergo chromosomal rearrangements. Genome compartmentalization was also identified in asexual lineages of the vascular wilt pathogen *Verticillium dahliae*, for which chromosomal rearrangements created extensive regions specific to asexual lineages of the pathogen. These lineage-specific chromosomal regions are enriched in genes that contribute to aggressiveness during host colonization (20). In smut fungi, several effector genes are organized in clusters flanked by transposable elements (21).

The functional compartmentalization of pathogen genomes raises intriguing questions regarding its origin and how repeat-rich genomic regions favor the evolution of virulence genes. A key step in the evolution of genome compartmentalization was shown to be the emergence of structural variation driven by transposable elements (20, 22). However, the emergence of compartmentalization through chromosomal rearrangements was until now only found among asexual lineages. Hence, structural variation was not constrained by homologous pairings of chromosomes during sexual reproduction. As structural variants suppress recombination rates, structural variants segregating in a sexual population are subject to reduced gene flow, reduced efficacy of selection, and ultimately, the accumulation of deleterious mutations. How genome compartmentalization could emerge in frequently recombining pathogen populations remains unresolved.

We used the highly polymorphic plant-pathogenic fungus *Zymoseptoria tritici* as a model to study incipient genome compartmentalization. *Z. tritici* is specialized to infect wheat and is among the most damaging pathogens of this crop (23). The fungus is pandemic, and populations are characterized by high levels of gene flow (24). Large effective population sizes (25) and high recombination rates (26) likely facilitated the rapid adaptive evolution observed over timescales of a few years. *Z. tritici* populations rapidly overcame resistance in wheat cultivars and repeatedly evolved resistance to multiple fungicide classes (27, 28). Despite the identification of both isolate-specific resistance and quantitative virulence (29), the genetic basis of the interaction between wheat and *Z. tritici* remains poorly understood (30).

The genome of *Z. tritici* contains 13 core chromosomes and up to 8 accessory chromosomes, which are highly unstable during meiosis and exhibit extensive structural variation in *Z. tritici* populations (31, 32). The *Z. tritici* genome is composed of 17% transposable elements, which are linked to variation in GC content along chromosomes (32, 33). Characterization of the genome and transcriptome of *Z. tritici* revealed a large number of putative effector genes encoding small secreted proteins (SSPs) that are likely involved in virulence (32, 33). Genes encoding SSPs evolved more rapidly than the genomic background (25, 34) and were more likely to lack orthologs in closely related species (33). Genomic analyses suggested that numerous genes (including effector genes) were deleted in at least some isolates of the species (31, 35).

In this study, we aimed to identify the genomic basis of structural variation in the species and investigate the role structural variants could play in the evolution of virulence. For this, we assembled a high-quality complete genome sequence of a highly virulent isolate to complement the completely assembled reference genome (32). Comparative genomic analyses between the complete, conspecific genomes enabled us to identify extensive structural variation, including large inversions and insertions. Homologous chromosomes frequently differed in the complete presence or absence of large clusters of transposable elements explaining the variations in chromosome length. The structural variation segregating within the species gave rise to hundreds of genes not shared between the two genomes. The genes not found in all members of the species were on a distinct evolutionary trajectory and were enriched in putative effector genes.

## RESULTS

### Complete fungal genome assembly.

We aimed to completely assemble the genome of the highly virulent Swiss *Z. tritici* isolate 3D7 (36). Isolate 3D7 was previously used to produce a mapping population for recombination rate and quantitative trait analyses (26, 37–39). We used 3.56 Gb of filtered single-molecule real-time (SMRT) sequencing data with a mean subread length of 5,632 bp to produce a draft assembly using HGAP3 (40). We evaluated the assembled contigs based on read coverage and removed contigs with excessively high or low coverage indicative of erroneous assemblies (see Fig. S1B in the supplemental material). The longest rejected contig was 25.8 kb in length. We retained 53 contigs totaling 38.3 Mb and a contig N50 of 2.78 Mb.

To validate and finish the genome assembly, we used the mapping population established previously for the same isolate (37). The single-nucleotide polymorphism (SNP) genotyping of the mapping population was based on restriction-associated DNA sequencing (RADseq) using Illumina short reads. We aligned the short-read data available for each progeny of the mapping population to the contigs of the 3D7 genome assembly for SNP calling. After filtering for quality and genotyping rate, we retained 245 progeny and 46,784 SNPs. We generated a linkage map comprising all SNPs called on the 3D7 contigs and obtained 17 linkage groups corresponding to the expected number of chromosomes (31). We found no evidence for misassembly, as all contigs were uniquely assigned to a single linkage group and the genetic and physical marker orders were highly correlated (see Fig. S1C in the supplemental material). Thirteen linkage groups were composed of a single contig, and four linkage groups were composed of 2 to 4 contigs each. Core chromosomes 1, 3 to 5, and 9 to 13 and all four accessory chromosomes (chromosomes 16, 17, 19, and 20) were each assembled as a single contig. For linkage groups composed of multiple contigs, we used the marker order within the linkage group to order and orient each of the contigs. Mapping the PacBio reads on the genome then successfully closed the gaps.

### Chromosomal length and inversion polymorphisms segregating within the species.

**FIG 1:**
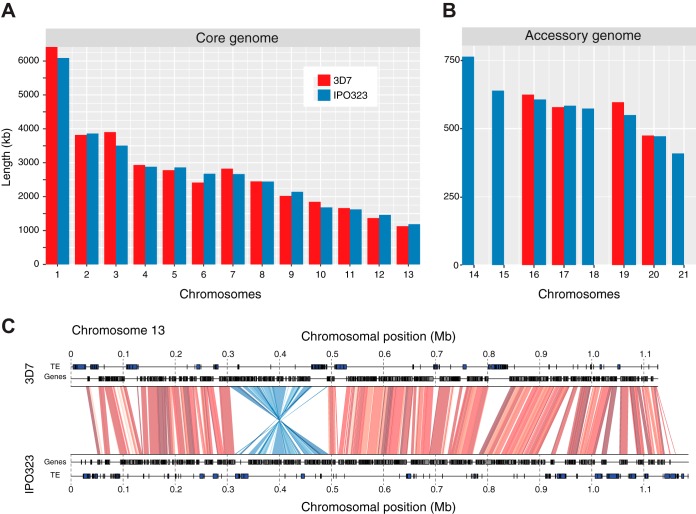
Chromosomal length variations between the 3D7 and IPO323 genomes of *Zymoseptoria tritici*. (A) Core chromosome length variation. (B) Accessory chromosome length variation. The genome of 3D7 lacks accessory chromosomes 14, 15, 18, and 21. (C) Large inversion polymorphism on the mating-type chromosome 13 shown by whole-chromosome synteny analyses. Collinear sequences between 3D7 and IPO323 are shown by red segments. Light to dark red shades indicate levels of sequence identity (90 to 100%). Inverted sequences are shown by blue segments, with shades of blue indicating levels of sequence identity (90 to 100%). For each genome, the locations of genes and transposable elements (TE) are shown as independent tracks.

The complete assembly of the 3D7 genome contained 17 chromosomes, and all chromosomes were homologous to chromosomes of the IPO323 genome (Fig. 1A and B). Isolate 3D7 lacked four accessory chromosomes (chromosomes 14, 15, 18, and 21), as previously reported (31), largely explaining the reduced genome size of 3D7 (37.9 Mb) compared to that of IPO323 (39.6 Mb). However, homologous chromosomes differed between 3D7 and IPO323 by 4.3 to 399 kb (see Table S1 in the supplemental material). Chromosomes 3, 6, and 10 were the most polymorphic, showing from 9.7 to 11.4% length variation compared to the homologous chromosomes of IPO323.

We screened the genome of 3D7 for chromosomal inversion compared to the genome of IPO323. We detected a total of 28 distinct inversions of at least 500 bp (see Table S2 in the supplemental material). Most inversions were located on the largest chromosome, chromosome 1 (*n* = 8). Inversions affected a total of 628 kb in the 3D7 genome, and a majority of inverted sequences (79%) contained at least one gene. In total, 211 genes were located on inverted sequences. The longest inversion spanned 189 kb, covered 17% of chromosome 13, and contained 65 genes (Fig. 1C). We detected large clusters of transposable elements at homologous positions in the inverted sequence. The inversion was located 142.7 kb from the mating type alpha-1 HMG box gene.

### Core and orphan gene content.

In order to determine whether chromosomal structural variation resulted in functional variation between the genomes, we predicted genes *de novo* in the 3D7 genome. We combined two types of evidence to guide the predictions, as follows: (i) we mapped protein sequences of isolate IPO323 (33) to the genome of 3D7, and (ii) we identified transcript locations using two deep-RNA-sequencing datasets generated for the complete infection cycle of 3D7 (41). We identified a total of 11,737 genes in 3D7. The numbers of genes predicted for the IPO323 genome were in a similar range (10,688 to 11,839) (32, 33, 42). A total of 7,353 predicted proteins (62.6%) contained at least one domain matching an entry in the Pfam database, and 1,034 proteins (8.8%) were likely to be secreted extracellularly (see Table S3 in the supplemental material). Plant pathogens secrete a large number of SSPs to modulate the host immune response. Hence, we used the machine-learning algorithm implemented in EffectorP (43) to identify genes most likely to encode effectors interacting with the host immune system. A screen of the secretome identified 330 proteins (2.8%) as strong candidates for SSPs interacting with the host (see Table S3). The proportions of both predicted secreted proteins and putative effector proteins were significantly larger in 3D7 than in IPO323 (Fisher exact test, *P* = 0.00494 and *P* = 0.0311, respectively).

Coevolution of plant pathogens with their hosts may lead to rapid gains or losses of pathogen genes if the encoded proteins either confer a strong benefit or the host immune systems evolve to detect them. Therefore, such genes may be located in genome compartments unique to individual genomes. A previous study identified reference genome genes which were likely missing in 13 sequenced Australian isolates (35). Using BLAST, we searched for genes in the IPO323 genome that were absent in the 3D7 genome. As the accessory chromosomes 14, 15, 18, and 21 were missing in 3D7, we excluded these from the analysis. We identified 216 genes that were missing in the 3D7 genome in comparison with the homologous chromosomes in IPO323 (see Table S4 in the supplemental material). Out of these 216 genes, 11 genes were located on accessory chromosomes.

**FIG 2:**
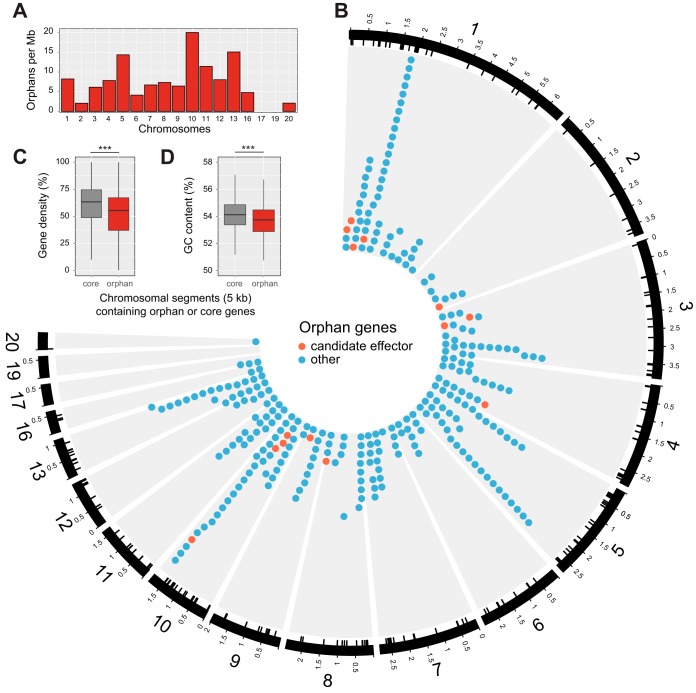
Genomic distribution of 296 orphan genes detected in the 3D7 genome. (A) Density of orphan genes across the 17 chromosomes of the 3D7 genome. Accessory chromosomes 17 and 19 contained no orphan genes. (B) Chromosomal locations of orphan genes. The outermost ring represents chromosomal positions in Mb. Internal tick marks show the exact location of orphan genes. Orphan genes are shown as circles according to chromosomal location. Orphan genes identified as effector candidates are shown in red. (C) Analysis of chromosomal segments containing at least one orphan gene or only core genes. Chromosomal segments containing only core genes were more gene dense than segments containing orphan genes (Wilcoxon rank sum, *P* < 0.0001). (D) Chromosomal segments containing orphan genes had lower GC contents (Wilcoxon rank sum, *P* < 0.0001).

To determine the extent and genomic localization of orphan genes in the 3D7 genome, we screened all 3D7 genes for homology in the completely assembled IPO323 genome using BLAST. We identified a total of 296 genes (2.5% of the transcriptome) without any homologous sequence. These intraspecific orphan genes represent genes previously unknown for the species. Among other proteins, these orphan genes encoded secreted peptidases, major facilitator superfamily proteins, glycosyl hydrolases, and a heterokaryon incompatibility protein (see Table S5 in the supplemental material). Orphan genes were found on all core chromosomes and two accessory chromosomes (Fig. 2A). The highest density of orphan genes was on chromosome 10, with 20 orphan genes per Mb (Fig. 2B). Accessory chromosomes had lower orphan gene densities than most core chromosomes. However, the number of orphan genes on accessory chromosomes was only 3, 0, 0, and 1 on chromosomes 16, 17, 19, and 20, respectively. Hence, a test for an under-representation of orphan genes on accessory chromosomes would not be meaningful.

**FIG 3:**
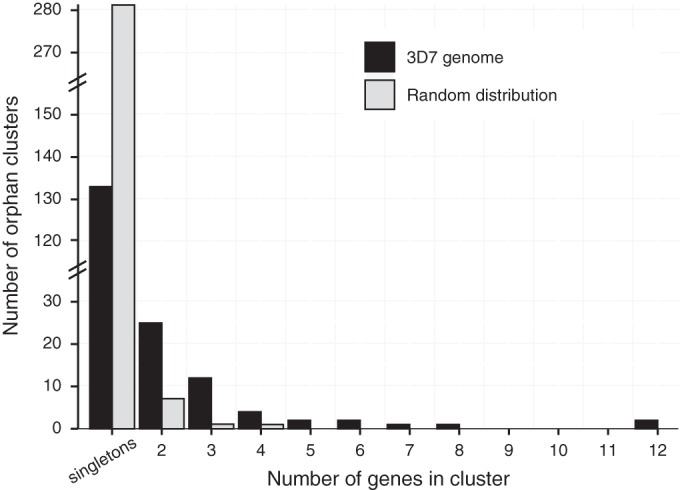
Clustering of orphan genes in the genome. The number of genes per orphan cluster is shown in comparison to the mean cluster size drawn from 1,000 randomly drawn distributions. Orphan genes were significantly more clustered than expected by chance (Fisher exact test, *P* < 0.0001). Standard errors from the randomly sampled orphan gene distributions were too small for visualization.

**FIG 4:**
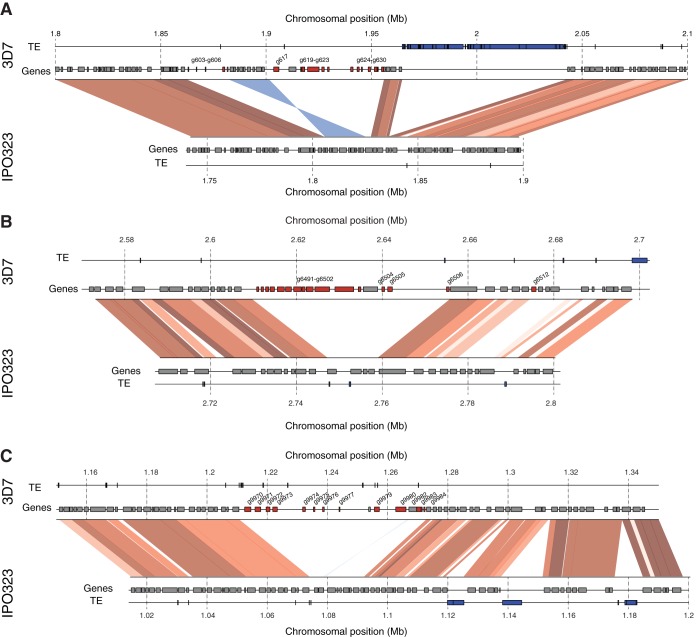
Chromosomal synteny analyses of regions harboring large orphan gene clusters. Collinear sequences between 3D7 and IPO323 are shown by red segments. Light to dark red shades indicate levels of sequence identity (90 to 100%). Inverted sequences are shown by blue segments, and shades of blue indicate sequence identity (90 to 100%). For each genome, the locations of genes and transposable elements (TE) are shown as independent tracks. (A) A cluster of 12 consecutive orphan genes (*ZT3D7_g619* to *ZT3D7_g630*) located on chromosome 1. An inversion polymorphism affecting 19 kb and 9 genes was located in proximity. The orphan gene cluster was also in proximity to a large TE cluster. (B) On chromosome 5, subtelomeric clusters contained 3 and 12 orphan genes, respectively. Gene *ZT3D7_g6501* encodes a peptidase C19. (C) On chromosome 10, a total of 13 orphan genes were clustered with interspersed core genes that have homologs in IPO323. Synteny between 3D7 and IPO323 was interrupted multiple times by nonsyntenic TE clusters.

Orphan genes were found in clusters significantly more often than expected by chance (Fisher exact test, *P* < 0.0001) (Fig. 3). We found clusters of 8, 12, and 12 orphan genes on chromosomes 1, 5, and 10, respectively. On chromosome 1, the 3D7 genome contained a 170-kb nonsyntenic region comprising a total of 17 orphan genes (Fig. 4A). The expanded region in 3D7 was characterized by a sequence inversion of 19 kb and a 43-kb insertion of a cluster of transposable elements. The subtelomeric region of chromosome 5 contained a cluster of orphan genes that was located at approximately 120 kb from the telomere (Fig. 4B). This cluster contained two subclusters of 3 and 12 genes, respectively, interspersed with a gene sharing weak homology in the IPO323 genome. The orphan gene *ZT3D7_g6501* encodes a ubiquitin-specific peptidase C19. A cluster with 13 orphan genes was found on chromosome 10 (Fig. 4C). These orphan genes were interspersed with two genes that shared weak homology in the IPO323 genome. Chromosome 10 of IPO323 was found to carry a number of transposable element clusters within 100 kb of the orphan gene cluster.

### Genomic environment of orphan genes.

**FIG 5:**
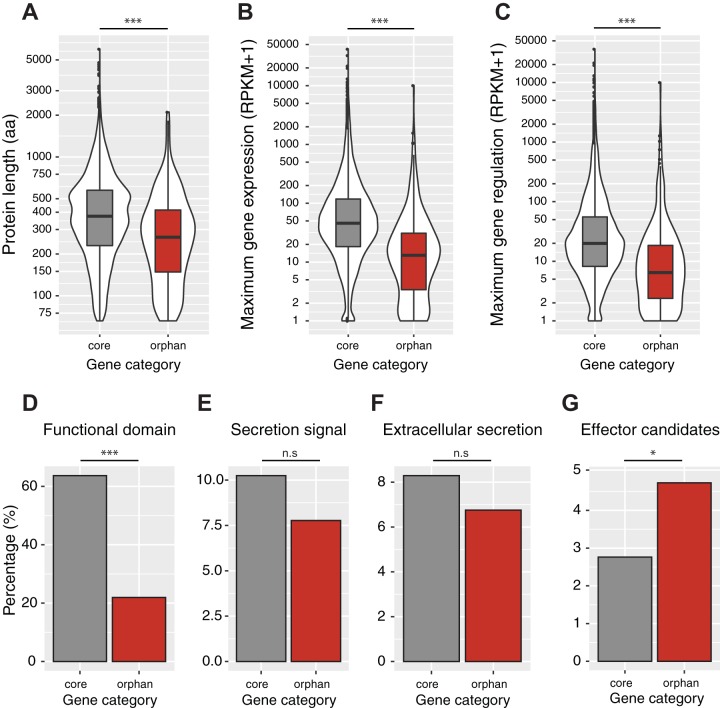
Differentiation of core and orphan genes in the 3D7 genome. (A) Orphan genes encoded significantly smaller proteins (Wilcoxon rank sum, *P* < 0.0001). (B and C) Orphan genes were significantly less transcribed during the infection of wheat leaves (B), and the difference between the minimum and maximum transcription levels was smaller (C) (Wilcoxon rank sum, *P* < 0.0001). (D) Orphan genes were less likely to encode a protein associated with a protein family (Pfam) domain (Fisher exact test, *P* < 0.0001). (E and F) Core and orphan genes were similarly likely to encode secreted proteins (E) and proteins targeted for extracellular secretion (F). (G) Orphan genes were more likely to encode candidate effectors based on classifications by EffectorP (43) (Fisher exact test, *P* = 0.0496).

We systematically analyzed chromosomal regions containing orphan genes and found that these genes were located in chromosomal compartments that were slightly but significantly less gene dense (Fig. 4C). Segments containing orphan genes showed slightly lower GC contents (Fig. 2D). Next, we investigated whether orphan genes differed functionally from core genes. We found that orphan genes encoded significantly shorter proteins and that orphan genes were transcribed significantly less on average (Fig. 5A and B). During infection, orphan genes were on average more weakly differentially regulated than core genes over the time course of an infection (Fig. 5C). Furthermore, orphan genes were significantly more likely to lack a functional domain based on Pfam annotations (Fig. 5D). Taken together, these characteristics suggest that orphan genes may be more affected by degeneration processes than core genes.

Orphan genes contributed disproportionally to the candidate effectors. We investigated whether orphan genes were encoding candidate effector proteins. For this, we analyzed whether genes were likely to encode secreted proteins and whether the amino acid sequence was characteristic of effector genes. We found that orphan genes did not encode proteins with significantly different proportions of secretion signals or predictions of extracellular localization (Fig. 5E and F). Then, we used EffectorP to identify the proteins most likely to be secreted effectors among all secreted proteins. We found that orphan genes were significantly enriched in genes predicted to encode effector proteins (Fig. 5G). Finally, we analyzed previously generated transcriptomics data over the entire time course of a wheat infection (41) to determine gene expression levels and identify differential regulation. The core secretome contained a large number of genes that were upregulated during infection (see Fig. S2 in the supplemental material). Proteins predicted to be effectors were shorter overall and contained more cysteines, consistent with current knowledge regarding the characteristics of fungal effectors (44) (see Fig. S2 and Table S6). The orphan secretome was substantially smaller than the core secretome, as discussed above (see Fig. S2). However, the orphan secretome contained highly upregulated effector gene candidates. We found that the orphan effector candidate ZT3D7_g9283 was the fourth-most-upregulated putative effector in the 3D7 genome (see Fig. S2).

**FIG 6:**
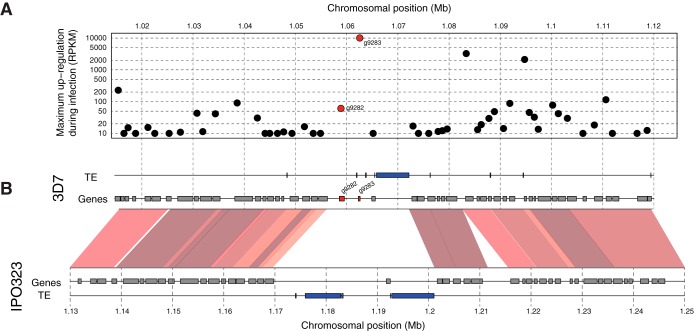
Chromosomal location of the most upregulated orphan gene during infection. The gene *ZT3D7_g9283* was predicted to encode an effector protein. (A) Maximum gene upregulation during wheat leaf infection in RPKM. Differential regulation was calculated among time points sampled 3 to 14 days postinoculation. (B) Chromosomal synteny analyses compared between 3D7 and IPO323. Sequences that were collinear between 3D7 and IPO323 are shown by red segments. Light to dark shades of red indicate levels of sequence identity (90 to 100%). For each genome, the locations of genes and transposable elements (TE) are shown as independent tracks. Orphan genes are indicated in red.

The effector candidate ZT3D7_g9283 encoded an 85-amino-acid protein that is highly enriched in cysteines (9.4%) and lacks homologs in other fungal species. We analyzed the chromosomal region in which ZT3D7_g9283 was located and found that the gene was the most-upregulated gene in a region of 100 kb during infection (Fig. 6A). The effector gene candidate ZT_3D7g9283 was adjacent to a second orphan gene (ZT3D7_g9282) not predicted to be an effector (Fig. 6B). The two orphan genes were located at the boundary between a gene-dense region that was conserved between IPO323 and 3D7 and a nonsyntenic chromosomal region containing transposable element (TE) clusters (Fig. 6B). The chromosomal region in 3D7 contained a single TE cluster within 3 kb of the orphan genes. In IPO323, the nonsyntenic region was expanded and contained two clusters of TEs.

## DISCUSSION

We used single-molecule real-time (SMRT) sequencing data and a high-density genetic map to completely assemble the genome of a highly virulent isolate of *Z. tritici*. Deep transcriptomic data available for the same isolate enabled high-quality gene predictions independently from gene models of the reference genome isolate IPO323. We discovered large-scale structural variation between the 3D7 and IPO323 genomes despite both isolates being members of the same sexually reproducing species. Breaks in synteny among the genomes included an inversion covering 17% of a chromosome. We found that nonhomologous regions of 3D7 harbored orphan genes not found in IPO323. Orphan genes were differentiated from core genes, showing hallmarks of degeneration that included shortened transcript lengths and weaker expression. However, the orphan genes were enriched in effector genes predicted to interact with the host immune system during infection.

We identified an extensive catalog of structural variation segregating within a sexually reproducing pathogen. Structural variation in fungal populations and species, including *Z. tritici*, was long suspected based on the abundant evidence for chromosomal length polymorphism (45–47). Our study demonstrated that completely assembled homologous chromosomes indeed differed in length. The length polymorphism among homologs was extensive enough to create differences in the rank order of chromosomal lengths between 3D7 and IPO323.

We found that chromosomal length variation was primarily caused by presence/absence polymorphisms of entire TE clusters that disrupted chromosomal synteny. In between TE clusters, long gene-dense tracts were structurally conserved. TE clusters lacking homologs during meiosis may locally depress the homologous recombination rate and also increase the likelihood of nonhomologous recombination. Hence, the organization of the chromosomes into gene-dense regions and gene-poor regions rich in transposable elements may be the result of selection acting to shield essential genes in gene-dense regions from the deleterious impact of nonhomologous recombination promoted by TE clusters.

Despite the abundance of inversion, insertion, and deletion polymorphisms, we did not find evidence for large-scale chromosomal translocation or fusion events as described among clonal lineages of the vascular wilt pathogen *V. dahliae* (20). Frequent sexual reproduction and high recombination rates in *Z. tritici* (26, 48) may enable efficient purging of deleterious chromosomal rearrangements. Comparative genomic analyses of *Z. tritici* and other Dothideomycetes showed that interchromosomal translocations were indeed very rare, despite the extensive reshuffling of gene order within chromosomes (49). Our findings of extensive intraspecific intrachromosomal structural variation but no large-scale translocations are consistent with a model of long-term persistence of gene localization on particular chromosomes. Structural variation segregating within species forms a powerful basis for rapid chromosomal diversification among species.

We identified 296 genes lacking any homologous sequence in the reference genome. We believe that the orphan gene count is a conservative estimate, as we filtered out any partial matches between the genomes and were identifying hits in the genomic sequence instead of relying on the quality of gene annotations. The robust identification of orphan genes also relied on comparing two complete genome assemblies. Gaps present in incomplete assemblies could easily lead to erroneously assigned orphan genes. The IPO323 genome was assembled telomere-to-telomere and validated by two genetic maps (32). The newly assembled 3D7 genome was similarly assembled with nearly complete telomeric ends and validated by genetic maps.

Orphan genes were not distributed randomly among chromosomes. Despite the extensive sequence variation found among homologous accessory chromosomes (31), we identified only four orphan genes (1.4%) located on these chromosomes. On core chromosomes, orphan genes tended to be organized in clusters of up to 12 consecutive genes, creating substantial nonsyntenic regions. In a sexually reproducing species, orphan regions are expected to undergo less recombination than highly collinear regions shared among all individuals (50). Reduced recombination rates affect the efficiency of selection acting on orphan regions and may trigger additional chromosomal rearrangements that would further decrease chromosomal collinearity.

Orphan genes showed hallmarks of relaxed selection and gene structure degeneration, as they were shorter and less transcribed. The largest orphan gene clusters were transcriptionally virtually silent. Decreased transcription levels may be caused by direct or indirect factors. Direct factors include reduced purging of deleterious mutations in promoter regions, leading to weakened transcriptional activation (51), while indirect factors can be related to the presence of TEs in orphan regions. The genomic defense mechanisms against TE activity in *Z. tritici* include silencing through changes in the chromatin state from euchromatin to heterochromatin, which are driven by posttranslational histone modifications (52). In *Z. tritici*, regions that are enriched in trimethylated histone H3 lysine 9 (H3K9me3) histone marks, a signature of heterochromatin, are strongly correlated with the presence of TEs (53). The second known genomic defense mechanism in *Z. tritici* is repeat-induced point mutation (RIP) (32). In Ascomycete species, RIP inactivates highly similar copies of sequences by introducing point mutations in CpG sites (15). Hence, the mutational load of RIP can rapidly degenerate gene structures. In summary, the association of orphan genes with TE can lead to a combined mutational load of nonhomologous recombination and gene silencing due to genomic defense mechanisms.

To trace the evolutionary fate of orphan genes in the species, we will require knowledge about the frequency of individual orphan genes in populations and processes affecting their inheritance. Rare orphan genes are less likely than frequent orphan genes to be matched during sexual reproduction and undergo homologous recombination. Large-scale population resequencing will be able to establish orphan gene variant frequencies and test predictions about levels of recombination rates and the impact on selection efficacy.

Over the past decade, pangenomic analyses of bacterial genomes have identified striking levels of genomic diversity among closely related strains (54). However, pangenomic analyses are in their infancy for complex eukaryotic genomes. The development of tools to integrate pangenomes into population genomics analyses remains a major challenge. Indeed, most population genomics analyses have been based on resequencing and variant identification compared to a single reference genome. Therefore, genetic variants identified in these studies were restricted to sequences present in the reference genome. Our study shows that such an approach can significantly underestimate diversity between genomes. Moreover, large-scale rearrangements in genomes can affect both recombination rates and linkage disequilibrium (50). Hence, structural variants can introduce biases in quantitative trait locus (QTL) and genome-wide association study (GWAS) analyses, as well as prevent the mapping of loci underlying phenotypic variation.

In many fungal genomes, genes involved in virulence show a pattern of presence/absence polymorphism among populations (22, 55–57). In the case of disease outbreaks caused by singular clones, focusing genomic analyses on the causal pathogen lineage is appropriate. However, in sexually reproducing pathogens, selection pressure can rapidly eliminate detrimental genes or sweep beneficial genes to fixation through recombination. Hence, the source of evolutionary innovation (e.g., a beneficial effector gene) can easily be missed when a single reference genome is used in population genomics analyses aimed at identifying genes under selection. The discovery of extensive orphan regions in populations of sexually reproducing species raises intriguing questions about the functional importance of such regions. A key question to address is how orphan regions arise in genomes and are maintained over evolutionary time. The role played by transposable elements will provide important clues to explain the emergence of genome compartmentalization (22).

## MATERIALS AND METHODS

### High-molecular-weight DNA extraction.

The Swiss *Z. tritici* strain ST99CH_3D7 (abbreviated 3D7 herein) was collected in 1999 from a Swiss wheat field and found to be highly virulent (36, 58). We used a modified version of the cetyltrimethylammonium bromide (CTAB) DNA extraction protocol developed for plant tissue (59). Fungal spores were lyophilized after 5 to 6 days of growth in liquid yeast sucrose broth (YSB). Approximately 60 to 100 mg of lyophilized spores were then crushed with a mortar. After the supernatant was transferred from the phenol-chloroform-isoamyl alcohol solution and centrifuged, the pellet was resuspended in fresh phenol-chloroform-isoamyl alcohol to repeat this step once. We performed the washing step three times. Finally, the pellet was resuspended in 100 µl of sterile water.

### Real-time single-molecule sequencing.

A PacBio SMRTbell library was prepared using 15 µg of high-molecular-weight DNA. The library was size selected with an 8-kb cutoff on a BluePippin system (Sage Science). After selection, the average fragment length was 15 kb. Sequencing was performed on two SMRT cells using P4/C2 chemistry and three SMRT cells using P6/C4 chemistry. PacBio sequencing was run on a PacBio RS II instrument at the Functional Genomics Center, Zurich, Switzerland.

### Genome assembly using self-corrected PacBio long reads.

PacBio read assembly was performed using HGAP version 3.0 included in the SMRTanalysis suite (version 2.3.0, patch 3) (40). HGAP was run using default settings except for the minimum seed read length to initiate the self-correction. The filtered SMRT sequencing data amounted to approximately 90-fold coverage of the expected genome size of 39 Mb (32). We tested minimum seed read lengths of 5,000, 6,000, 7,000, 8,000, 9,000, and 9,218 bp, with the last length being the cutoff automatically chosen by HGAP. We evaluated the effects of the minimum seed read lengths on the preassembly yield (successfully preassembled bases versus the total seed bases entering preassembly), the assembly N50, and the total assembly length. We retained the 6,000-bp minimum seed read length cutoff. The assembled contigs were polished using Quiver with default settings as implemented in the SMRTanalysis suite.

We identified problematic contigs in the assembly by analyzing the coverage of PacBio read alignments on each contig. We discarded contigs if the median coverage of a contig deviated by more than a factor of 1.5× from the median coverage of all contigs weighted by contig length.

### Assembly validation using a high-density genetic map.

We used high-density genetic maps to validate the contiguity of HGAP-assembled contigs and to finalize the assembly of contigs into chromosomes. For this, we used the previously generated restriction-associated DNA sequencing data of a progeny population produced from a cross between isolates 3D7 and 3D1 (isolated from the same wheat field) (37). We aligned quality-filtered Illumina reads obtained from each progeny in the cross to HGAP-assembled contigs using Bowtie2 (60). SNP calling was performed, first individually per progeny using HaplotypeCaller implemented in the Genome Analysis Toolkit version 3.3 (61), using settings for a haploid genome and setting the maximum number of alternative alleles to 1. Then, SNP calls were combined and evaluated for the entire progeny population using the GenotypeGVCFs tool. We filtered SNPs by failure to meet the following criteria: QUAL >5,000, QD >5, MQ >20, and ReadPosRankSum, MQRankSum, and BaseQRankSum between −2 and 2. The filter criteria were selected primarily to remove SNPs called from ambiguously mapped reads.

High-quality SNPs and progeny were filtered for genotyping rates. First, we removed all progeny with less than 75% of SNPs being called, and second, we removed all SNPs with a genotyping rate below 90% among the retained progeny. A genetic map was constructed using MSTmap implemented in the R package (62, 63). We chose the Kosambi distance function and a *P* value cutoff of 1e−6. Genetic maps linking individual contigs into chromosomes were visually inspected for continuous gradients of genetic distances and physical distances, evidence for contig misassemblies (a contig associated with more than one linkage group), and contig orientation within the linkage group (based on the genetic marker order).

The linkage group information was used to construct chromosomal sequences from individual contigs. If a linkage group contained more than one contig, individual contigs were joined in the order and orientation as defined by the genetic map. Gaps (N) were introduced to maintain spacing between individual contigs within chromosomes.

### Gap filling and assembly polishing.

For chromosomes consisting of more than one contig, we attempted to span and fill gaps using PBJelly version 15.8.24 (64). All PacBio reads generated for isolate 3D7 were used for gap filling. Contigs were only allowed to be joined if the contigs were located at adjacent positions within a linkage map (--capturedOnly option in PBJelly). Gaps filled by PBJelly were subsequently error corrected using Quiver (SMRTanalysis suite). A final polishing step was performed using PILON (65) and Illumina short read data generated for 3D7 in a previous study accessible from the NCBI Short Read Archive under accession number SRS383147 (31). PILON was set to correct indels and SNPs detected by aligning Illumina reads to the assembled chromosomal sequences.

### Repetitive elements annotation.

We annotated repetitive elements in the genome of 3D7 using RepeatModeler version 1.0.8 (A. F. A. Smit and R. Hubley, RepeatModeler Open-1.0 2008-2015; http://www.repeatmasker.org). RepeatModeler includes both RECON and RepeatScout for the *de novo* prediction and modeling of repeat families using rmblast. *De novo* repeat families and repeat families known from the RepBase database were classified and annotated. Finally, the genome of 3D7 was masked using RepeatMasker version 4.0.5 (A. F. A. Smit, R. Hubley, and P. Green, RepeatMasker Open-4.0 2013-2015; http://www.repeatmasker.org) for synteny analyses and gene model predictions.

### RNA-seq-assisted gene model prediction.

The accuracy of gene prediction is significantly enhanced by incorporating evidence for intron splice sites and gene models from a closely related organism (66). The transcriptome of 3D7 was previously characterized using transcriptome sequencing (RNA-seq) (41). We used RNA-seq data covering six time points throughout the infection cycle of 3D7 on wheat seedlings (3 to 56 days postinfection [dpi]). We also used additional deep RNA-seq data generated for time points of 7, 14, and 56 dpi. All raw data were accessed from the records deposited in the NCBI Short Read Archive under accession numbers SRX1116288 and SRX1116289. RNA-seq reads were quality trimmed using Trimmomatic version 0.33 (67) and aligned to the 3D7 genome using TopHat version 2.0.14 (68). Intron splice site hints were generated using bam2hints, included in the AUGUSTUS version 3.2.1 software distribution (69). Due to the very high RNA-sequencing depth available, intron splice hints were filtered for a minimum coverage of 20 reads to avoid an impact of spurious splice signals on gene prediction.

In addition to splice site hints, we mapped predicted protein sequences of the fully sequenced reference genome of *Z. tritici* isolate IPO323 to the genome of 3D7 using exonerate (protein2genome model; minimum identity 95%) (70). We retrieved protein sequences based on the RNA-seq-assisted and significantly improved gene models by Grandaubert et al. (33).

We used the BRAKER version 1.0 pipeline (71) combining GeneMark-ET *ab initio* gene model predictions and AUGUSTUS version 3.2.1. GeneMark-ET was trained using the RNA-seq-based splice information as hints and produced *ab initio* gene models. AUGUSTUS was automatically trained using *ab initio* gene models that were fully supported by splice information. Finally, AUGUSTUS was used to predict gene models using both RNA-seq splice information and coding sequence hints based on exonerate protein alignments as extrinsic evidence.

### Functional annotation of genes.

We used InterProScan version 5.16-55.0 (72) to functionally annotate gene models. We assigned protein sequence motifs to protein families (Pfam) and gene ontology (GO) terms based on hidden Markov models (HMM) implemented in InterProScan. Furthermore, we screened protein sequences for the presence of secretion signal, transmembrane, cytoplasmic, and extracellular domains using a combination of SignalP version 4.1 (73), Phobius version 1.01 (74), and TMHMM version 2.0 (75).

### Differential gene expression analyses during infection.

We assessed the transcription profiles of all genes using RNA-seq data generated from the 3D7 isolate (41). Plants of the wheat cultivar Drifter were infected, and leaves were collected between 3 and 56 days postinfection (dpi). Time points between 3 and 14 dpi cover both the early asymptomatic and late symptomatic infection phases (41). Time points from 21 to 56 dpi were considered to be the saprotrophic growth phase of the fungus. We mapped raw reads retrieved from the data deposited in the NCBI Short Read Archive under accession number SRX1116288. We quality trimmed reads using Trimmomatic version 0.33 (67), using the settings “ILLUMINACLIP:TruSeq3-PE.fa:2:30:10 LEADING:10 TRAILING:10 SLIDINGWINDOW:5:10 MINLEN:50.” We used TopHat version 2.0.14 to align reads to the 3D7 genome and included the genome annotation as a guide (68). Mapped reads overlapping gene models were counted using HTSeq-count, setting the matching mode to “union” and filtering out reads with an alignment quality below 10 (76). Reads per kilobase of transcript per million mapped reads (RPKM) were calculated using the R package edgeR (77). The maximum upregulation of genes during infection was calculated by identifying the maximum and minimum RPKM among the time points covering the infection (3, 7, 11, and 14 dpi).

### Prediction of the secretome and identification of candidate effectors.

We defined the predicted secretome as all proteins satisfying the following criteria: presence of a secretion signal predicted both by SignalP and Phobius, absence of transmembrane domains predicted both by Phobius and TMHMM, and presence of an extracellular domain and absence of a cytoplasmic domain according to Phobius. We used the predicted secretome to identify the most likely effector candidates, filtering for protein length, cysteine content, and differential regulation during the infection phase. Additionally, we identified the most likely effector candidate pool from the secretome using the machine-learning approach implemented in EffectorP version 1.0 (43). EffectorP was initially trained on functionally confirmed effector genes in different plant-pathogenic fungi. See Sperschneider et al. (43) for details on the effector gene library used for training.

### Orphan genes and chromosomal synteny analyses.

The predicted genes in 3D7 were mapped on the genome of IPO323 using BLASTn version 2.4.0 (78). The orphan genes were defined as having no blast hit in the IPO323 genome. Synteny analyses between the 3D7 and IPO323 genomes were performed using BLASTn on repeat masked genomic sequences. As the synteny analyses were performed for intraspecific genome comparisons, blast hits were stringently filtered for a minimum alignment length of 500 bp, a minimum identity of 90%, and an E value of 0. Syntenic regions were visualized jointly with the location of genes and transposable elements using the R package genoPlotR (79).

### Accession number(s).

The genome assembly and annotation of the 3D7 genome were submitted to the European Nucleotide Archive (http://www.ebi.ac.uk/ena) and are available under accession number PRJEB14341.

## SUPPLEMENTAL MATERIAL

Figure S1  Genome assembly and validation procedure. (A) Flow chart of the genome assembly and annotation pipeline. See Materials and Methods for a detailed description of each stage. (B) Contigs assembled from self-corrected PacBio single-molecule real-time (SMRT) sequencing data were screened for mean read coverage. Contig coverage is an indicator for collapsed repeats or weak links. Contigs with coverage deviating from the expected mean (shown in red) were excluded from further assembly stages. (C) Validation of the chromosomal assembly by genetic maps. Correlation of genetic distances and physical distances on the complete assembly of chromosome 1. Genetic maps were estimated using RADseq markers genotyped in a cross between isolates 3D7 and 3D1. Download Figure S1, EPS file, 1.5 MB

Figure S2  Core and orphan genes encoding secreted proteins. Core genes (with homologs in the IPO323 genome) and orphan genes (lacking homologous sequences) were analyzed for upregulation during different stages of a wheat leaf infection (3 to 14 days postinoculation). Encoded proteins were characterized according to protein length and percentage of cysteines in the amino acid sequence. Candidate effector proteins are highlighted in red. The orphan *ZT3D7_g9283* is the fourth-most up-regulated effector candidate gene in the genome. Download Figure S2, EPS file, 2.5 MB

Table S1  Chromosomal length variations between the newly assembled 3D7 genome and the IPO323 reference genome. Isolate 3D7 is missing the accessory chromosomes 14, 15, 18, and 21. Table S1, XLSX file, 0.1 MB

Table S2  Chromosomal inversions segregating within the species. The locations of detected inversions between the 3D7 and IPO323 genomes are reported for each genome individually, including their length, and the number of genes affected in the 3D7 genome. Table S2, XLSX file, 0.1 MB

Table S3  Gene annotation of the 3D7 genome. Coordinates, annotation, and *in planta* expression values (RPKM, reads per kilobase of transcript per million mapped reads) of the genes predicted *de novo* in the 3D7 genome. The correspondence of these gene models with the gene models predicted in IPO323 was assessed by BLAST. Table S3, XLSX file, 2.8 MB

Table S4  Genes of isolate IPO323 that are missing in isolate 3D7. The nonhomologous accessory chromosomes 14, 15, 18, and 21 are missing in 3D7 and were omitted from the analyses. Table S4, XLSX file, 0.1 MB

Table S5  List of orphan genes detected in the 3D7 genome. Coordinates, annotation, and *in planta* expression values (RPKM, reads per kilobase of transcript per million mapped reads) of the orphan genes identified in the 3D7 genome. The correspondence of these gene models with the gene models predicted in IPO323 was assessed by BLAST. Table S5, XLSX file, 0.1 MB

Table S6  Orphan genes predicted to encode effector proteins in the 3D7 genome and their expression *in planta* Table S6, XLSX file, 0.1 MB
